# Clinical and Laboratory Diagnosis of Buruli Ulcer Disease: A Systematic Review

**DOI:** 10.1155/2016/5310718

**Published:** 2016-06-20

**Authors:** Samuel A. Sakyi, Samuel Y. Aboagye, Isaac Darko Otchere, Dorothy Yeboah-Manu

**Affiliations:** ^1^Department of Bacteriology, Noguchi Memorial Institute for Medical Research, University of Ghana, Accra, Ghana; ^2^Department of Molecular Medicine, School of Medical Sciences, Kwame Nkrumah University of Science and Technology (KNUST), Kumasi, Ghana; ^3^Department of Biochemistry, Cell and Molecular Biology, University of Ghana, Accra, Ghana

## Abstract

*Background.* Buruli ulcer (BU) is a necrotizing cutaneous infection caused by* Mycobacterium ulcerans.* Early diagnosis is crucial to prevent morbid effects and misuse of drugs. We review developments in laboratory diagnosis of BU, discuss limitations of available diagnostic methods, and give a perspective on the potential of using aptamers as point-of-care.* Methods.* Information for this review was searched through PubMed, web of knowledge, and identified data up to December 2015. References from relevant articles and reports from WHO Annual Meeting of the Global Buruli Ulcer initiative were also used. Finally, 59 articles were used.* Results.* The main laboratory methods for BU diagnosis are microscopy, culture, PCR, and histopathology. Microscopy and PCR are used routinely for diagnosis. PCR targeting* IS2404* is the gold standard for laboratory confirmation. Culture remains the only method that detects viable bacilli, used for diagnosing relapse and accrued isolates for epidemiological investigation as well as monitoring drug resistance. Laboratory confirmation is done at centers distant from endemic communities reducing confirmation to a quality assurance.* Conclusions*. Current efforts aimed at developing point-of-care diagnostics are saddled with major drawbacks; we, however, postulate that selection of aptamers against MU target can be used as point of care.

## 1. Introduction

Buruli ulcer disease (BUD) is a neglected tropical disease caused by the environmental pathogen* Mycobacterium ulcerans* (MU). The disease is characterized by necrotizing, ulcerative lesions of subcutaneous fat and the overlying skin and is prevalent in poor regions of Africa, the Americas, Asia, and the Western Pacific [1]. The exact mode of transmission of MU remains unclear, but accruing data suggests that, probably, different modes of transmission occur in different geographic areas and epidemiological settings [2]. BUD begins with a preulcerative stage characterized by a firm nontender nodule, edema, or plaque with large areas of indurated skin, which is then followed by ulceration due to extensive skin cell destruction leading to the typically undermined edges [3, 4]. If left untreated, self-healing may occur which often leads to loss of vital organs and contractures. Even though mortality is low, morbidity and subsequent functional disability can be severe [5–8]. The main virulence factor responsible for the pathology of BUD is mycolactone. Mycolactone, an immunosuppressive and cytotoxic macrocyclic polyketide, is widely distributed within infected human lesions and has been postulated as a marker for diagnosis of BUD [9]. The social and economic burden of BUD can be high, particularly in impoverished rural regions. The disease affects both sexes equally and all age groups, but it is particularly common in children under the age of 15 [10].

Previously, BUD was treated by wide surgical excision followed by skin grafting; however, a study initiated by WHO and conducted in Ghana indicated that BU lesions can be sterilized by treatment with streptomycin and rifampicin [11]. Following that, the mainstay treatment protocol for BU is daily oral rifampicin plus intramuscular injection of streptomycin for 56 days, reducing surgery as an adjunct for correction of deformities [3, 12]. With the introduction of this antimycobacterial treatment, confirmation of clinically suspected cases is even more crucial for the clinical management of BU to prevent misdiagnosis and hence administration of unnecessary antibiotics. Previous reports of individuals treated for BU but were later found not to be BU by laboratory confirmation are available in literature [13–15].

Laboratory diagnosis of BU is multifaceted and has evolved over the years. There are currently four main methods that are being used for the laboratory confirmation of BUD and include microscopy for detecting acid-fast bacilli, culture to isolate viable organism, PCR for detecting pathogen specific DNA which is usually* IS2404*, and histopathology. The WHO recommends two laboratory tests to confirm BUD. However, in endemic settings, one may consider one positive test result from PCR or microscopy appropriate for the confirmation of clinical diagnosis because of the high positive predictive values for PCR (100%) and microscopy (97%) [3, 16]. In this review, we describe developments in the field of laboratory diagnosis of BUD, discuss applications and limitations of currently available diagnostic methods, and provide data on positivity and sensitivity ratios. This review further gives a perspective on the potential of selecting aptamers against MU targets for the development of a point-of-care diagnostics for BUD.

## 2. Methods

### 2.1. Search Strategy and Selection Criteria

Searched information for this review was done through PubMed, web of knowledge and Embase databases, and identified data up to December, 2015. References from relevant articles together with other published data from the WHO website and unpublished data presented at annual WHO advisory group meetings on Buruli ulcer were also used. The literature search was done using the following keywords:* Mycobacterium ulcerans*, laboratory diagnosis and confirmation, and methods for BU diagnosis and BU.

### 2.2. Assessment and Data Extraction

**Figure 1:**
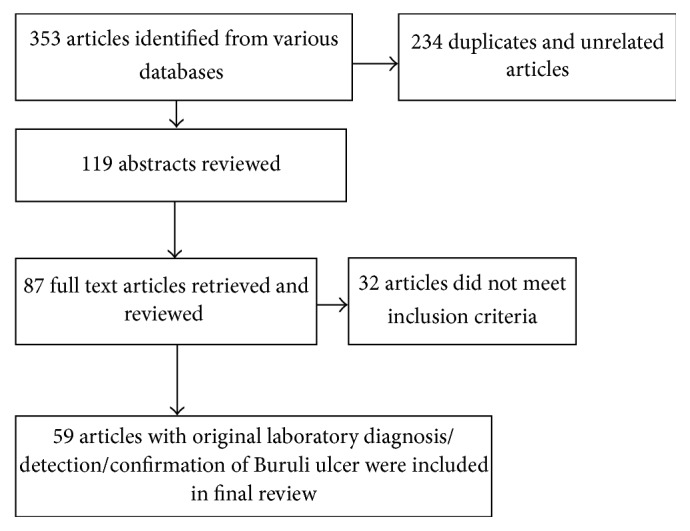
Schematic selection of review articles.

Articles in the full-text review were classified as containing original laboratory diagnostic methods for Buruli ulcer including sample collection methods, microscopy, culture, molecular techniques (PCR and its offshoots), and histopathology.  Figure 1 illustrates how the review articles were searched and selected.

## 3. Results and Discussion

### 3.1. Samples for Laboratory Confirmation of Buruli Ulcer

Samples for laboratory diagnosis of BUD include swabs and tissue specimens [17] from punch biopsies, surgical excision [18], and fine needle aspirates (FNA) [19]. FNA and tissues are used for analysis of nonulcerative lesions, whilst all other specimen types can be collected from ulcerative tissues [20]. However, with the advent of chemotherapy, FNA and swabs are becoming the preferred sample for laboratory confirmation. Recommendations for sample collection include the following: (1) swabs should be collected by circling the entire undermined edge of ulcerative lesions to maximize cell collection as MU is not uniformly distributed in the ulcers [21]. A good sample collection can be achieved through collection of at least two swabs per lesion; (2) FNA should be collected from the weakest part of the lesion to increase the chance of collecting MU cells; and (3) tissue samples from ulcerative lesions should be taken from the edge of the lesion, preferably below the end of the undermined edge, and should contain necrotic tissue. For nonulcerative lesions, tissue samples should be collected from the center of the lesion. Tissue samples must always contain subcutaneous adipose tissue. All the samples including FNA should be evaluated by microscopy, PCR, and cultivation [22, 23]. Laboratory confirmation of osteomyelitis cases requires whole bone samples (e.g., from amputation specimens) or curetted bone samples [17, 24, 25].  Table 1 summarizes the various types of specimen and transport media used for diagnosing BUD.

### 3.2. Microscopy

Microscopy is a quick, comparatively simple, and low-cost approach for the laboratory confirmation of suspected BUD cases and can be done with FNA, tissue, or swabs specimen. Microscopic diagnoses by direct smear examination with Ziehl-Neelsen staining to detect the presence of acid-fast bacilli are done using the quantification of smears in accordance with the method locally used for the diagnosis of TB [25]. The technological simplicity and requirement of low infrastructure allow microscopy to be conducted at all levels of health care delivery, even in less resourced countries. However, recorded sensitivity in literature is quite low and therefore undermines the overreliance of microscopy for case confirmation. Studies in Ghana and Benin which used microscopy as a first-line diagnosis of BU reported positivity rates between 40% and 78% [24, 37, 40].

Tissue smears prepared from ground samples can also be used for microscopy as well as from material hitherto subjected to decontamination procedures for culture. Nevertheless, according to a recent study in Benin, grounding of tissue does not increase the sensitivity of tissue smears (56.7%) compared with direct smears prepared from unground tissue (sensitivity, 59.4%) [40]. Whilst ZN staining is used in most of the studies, some other studies have suggested that Kinyoun and auramine-rhodamine staining techniques can also be applied to MU [8, 40].

### 3.3. Cultivation of* Mycobacterium ulcerans* from Clinical Specimen

Isolation of viable MU by culture is the final proof method among the diagnostics; however, due to the technological and infrastructure demand such as biosafety cabinets, cultures are done mainly at research centers of endemic and northern countries. Cultivation of MU from swabs and punch biopsies is normally transported in Middlebrook 7H9 broth supplemented with polymyxin B, azlocillin, amphotericin B, nalidixic acid, and trimethoprim (PANTA, Becton Dickinson Biosciences, NJ, USA). Additional supplementation with 0.5% agar yields a semisolid transport medium (STM) and preserves positive samples for up to 21 days [25, 41]. Although a number of culture media have been evaluated [34, 42, 43], Lowenstein-Jensen is considered the most appropriate medium for MU [42, 44]. Cultures are typically positive within 9–12 weeks of incubation at 29–33°C. Yet still, longer incubation times of up to 9 months have been observed [8]. Culturing MU from clinical samples is difficult and has a low sensitivity of about 35–60% [45]. The bacteria are extremely slow growing (6–8 weeks) and culture media are repeatedly contaminated with other faster growing species [7, 12, 26, 46]. This makes cultures unsuitable for quick laboratory confirmation and is limited to laboratory facilities with class II safety cabinets. The contamination effect of fast growing species are, however, counteracted by decontaminating the sample with either an acid and or a base to remove the unwanted fast growers using protocols such as the modified Petroff method (sodium hydroxide) [8], and the reversed Petroff technique (“Fortep” technique) [44]. In a decontamination protocol study conducted in Ghana, three different decontamination procedures were evaluated and concluded that a simple oxalic acid decontamination method produces high recovery rates [26, 34].

Notwithstanding these drawbacks, cultures are considered the only currently available valid confirmatory test for detection of viable bacilli in clinically suspected relapses and patients with nonhealing lesions after antimycobacterial treatment [24]. Furthermore, cultures are required for speciation, susceptibility testing, and other downstream applications [41]. Culture positivity ratios of 3–80% and sensitivities of 45–70% have been reported [6, 37, 46, 47]. The isolation of acid-fast bacilli from BUD patients alone does not offer adequate proof of the presence of MU. A cohort study in Ghana, indicated that a number of patients harbor other nontuberculous mycobacteria [37]. It is thus imperative that a confirmation of cultured isolates should be done. The main methods that have been used for isolate confirmation include sequence analysis and/or PCR detection of the insertion sequences* IS2404*,* IS2606*, ketoreductase gene of the giant plasmid,* rpoB* gene, the 16S–23S ribosomal RNA (*rRNA*) internal transcribed spacer gene, the 16S* rRNA* gene, VNTR, and the 65-kDa* hsp* gene, [17, 48–51].

### 3.4. Histopathology

**Figure 2:**
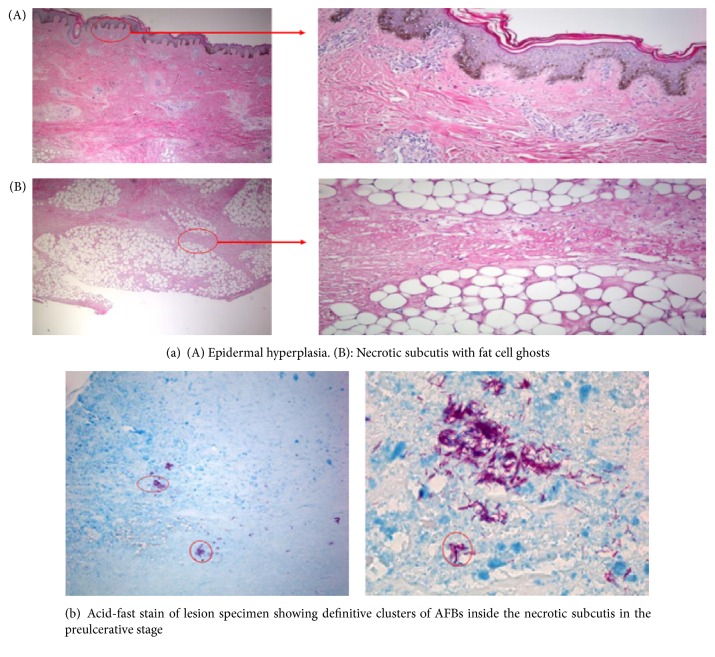
Histopathological images of Buruli ulcer disease.

Histopathology as a diagnostic method for BUD provides a fairly rapid result with a very high sensitivity (about 90%) [25]. It is also useful in establishing differential diagnosis and monitoring response to treatment. Histopathological analysis is carried out on tissue specimens in 10% neutral or buffered (pH 7.4) formalin stained with hematoxylin and eosin, Ziehl-Neelsen, or Kinyoun, and auramine-rhodamine. Distinctive histopathological features of BUD comprise the presence of acid-fast bacilli, (AFB) hyperplasia of the epidermis, elastolysis, inflammation, vascular variations of the dermis, and fat necrosis of the subcutis [25, 44]. In nonulcerated lesions, the epidermis is unbroken but hyperplastic. The upper dermis is intact but shows several stages of degeneration with infiltration of inflammatory cells. There is also clotting necrosis of the lower dermis, subcutaneous tissue, and underlying fascia with oedema. Vasculitis is common in the subcutaneous tissue. The ZN stain reveals large numbers of extracellular AFB in clusters, confined to the necrotic areas. In ulcerative lesions, ulcers are undermined with reepithelialization of the edges of the lesion and undersurface of the superimposing flap of the dermis. Neighboring epidermis is usually hyperplastic with AFB located at the base of the central slough and necrotic subcutaneous tissue [25]. Many studies have suggested that histopathology can identify about 30% additional cases than other confirmatory tests combined, mainly from paucibacillary late or healing stages of the disease [20, 24, 47, 52]. However, histopathological features cannot always provide clear-cut identification, as granulomas diffuse mixed cellular infiltrates and dense lymphocyte aggregates in the locality of vessels during antibiotic treatment [53]. Moreover, the method is expensive to perform and requires a sophisticated laboratory and highly trained personnel. Furthermore, the technique is invasive as it requires 3 mm to 4 mm in diameter punch biopsies.  Figure 2(a)(A and B) indicates epidermal hyperplasia and necrotic subcutis with fat cell ghost, respectively, whilst Figure 2(b) indicates acid-fast stain of lesion specimen showing characteristic clusters of AFB in the preulcerative stage.

### 3.5. Polymerase Chain Reaction (PCR)

Polymerase chain reaction (PCR) methods have been developed for BU diagnosis based on the insertion sequence* IS2404* [54], 16S* rRNA* gene [45], and the* hsp*-*65* gene [55]. The most routinely used PCR methods are conventional single-step gel-based PCR and real-time PCR targeting the insertion element* IS2404*. The insertion sequence* IS2404* is present in high copy numbers in the MU genome and it is considered as the gold standard because it has the highest sensitivity [56] and results are accessible within a short time. A positive PCR result is considered sufficient evidence to commence antimycobacterial treatment; moreover, real-time PCR is being considered for monitoring antimycobacterial treatment. However, the technique is expensive, requires sophisticated laboratory, and expertise, a strict quality control, and does not distinguish between viable and nonviable organism [3, 57]. A WHO report further encourages endemic countries to confirm at least 50% of all cases of PCR, either locally or with an external PCR reference laboratory [16].

DNA extraction is a crucial step in PCR processes and different methods involving in-house as well as commercial kits are being used. Methods involving mechanical homogenization in a digestion buffer followed by proteinase K digestion and purification by the guanidinium thiocyanate-diatoms methods have been applied successfully [29, 58]. Durnez et al. compared two adapted extraction methods, the modified Boom (MB) DNA extraction procedure with a commercial Maxwell® 16 DNA extraction procedure (M16, Promega, WI, USA), based on enzymatic lysis and paramagnetic separation, and demonstrated the superiority of the MB in terms of* IS2404* PCR sensitivity with clinical samples [38]. Another study compared semiautomated DNA extraction method using Maxwell kit with a modified Boom method and observed that Maxwell extraction method, performed on nondecontaminated suspensions, is the best for the molecular diagnosis of MU [39]. Other promising methods include heat and alkaline lysis by NaOH and sodium dodecyl sulphate followed by phenol-chloroform purification [26, 34, 59]. Many commercially available kits particularly Gentra systems and Puregene Genomic DNA purification kits have successfully been used with proteinase k to extract DNA from swabs, FNA, and tissue samples [17, 24, 44, 47]. It is recommended that DNA extraction is performed in a separate area using dedicated reagents and equipment to reduce the possibility of contamination.

Samples for PCR can be processed within hours to a day without prior storage in transport media [8] or stored at −20°C until processing or stored in transport buffers which is compatible with the extraction method [29, 58]. Many studies used transport media enriched with OADC, supplemented with PANTA and 0.5% agar [22, 42, 48, 57, 60, 61]. Transport of samples in liquid nitrogen has also been reported [21], dried swabs are also being used for DNA extraction, and positive PCR has been achieved after two weeks. PCR can also be done on paraffin-embedded tissue specimens using xylene-based deparaffinization for 10 minutes at room temperature [16, 54].

The initial primer design used for detecting MU insertion sequence* IS2404* was MU1 and MU2 for amplification of a 569 bp fragment. These primers were burdened with spurious banding and were improved with MU5 and MU6 primers which amplify the 492 bp fragment [54]. This was tested with a panel of 45 mycobacteria and other organisms and obtained 100% specificity and detection sensitivity of at least 0.1 genome equivalents [59, 62, 63]. Primers used in nested* IS2404*-based PCR include MU1 and MU2 for amplification of a 569 bp fragment of* IS2404* and PGP3 and PGP4 for amplification of a 217 bp product [32, 57]. Primers PU4F and PU7Rbio with a modified PCR protocol for amplification of a 154 bp product of* IS2404* have also been described [29, 58]. For real-time PCR, TaqMan primer sequences are mostly used.

Most endemic countries are tropical and hence the development of a dry reagent based PCR (DRB-PCR) which uses lyophilized reagents (PuReTaq Ready-To-Go-Beads, Amersham, UK) and primers have been employed to simplify the process and reduce incidence of false positives [47, 56] and requirement for elaborate infrastructure for PCR. Specific real-time PCR assay allows quantitative valuation and distribution of MU in BUD lesions and has exhibited much higher sensitivity than the conventional single-run gel-based* IS2404* PCR. Moreover, the enhanced TaqMan real-time PCR assay shows 12.5% higher diagnostic sensitivity compared with cultures; the assay reduces contamination and turnaround times for diagnosis and has been used routinely in Australia [61, 64]. Fyfe et al. developed two TaqMan Multiplex real-time PCR assays targeting three independent repeated sequences in the* M. ulcerans* genome, two multicopy insertion sequences (*IS2404*,* IS2606*), and a multicopy sequence encoding the ketoreductase B domain (KR-B) [22]. Affolabi et al. compared a single-step PCR, a nested PCR, and a real-time quantitative PCR on 74 surgical specimens from patients with clinically suspected Buruli ulcer and observed that real-time PCR after the modified Boom extraction method and a single-run PCR assay after the Maxwell extraction method, performed on nondecontaminated suspensions, are the best for the molecular diagnosis of BUD [39]. Guimaraes-Peres et al. assessed two nested PCRs, the nested* IS2404*-based PCR and the nested 16S rRNA gene-based PCR, and observed that the 16S rRNA gene-based PCR was positive for both MU and* M. marinum*; they suggested that the use of* IS2404-*based PCR showed better specificity, required less time, and was less costly than the 16S rRNA gene-based PCR [57]. Stienstra et al. also evaluated the* IS2404*-based nested PCR to detect MU from 143 BUD patients in Ghana. They further compared it with culture and histopathology results and recommended that small tissue samples might be sufficient for case confirmation in future studies [32]. Phillips et al. also used* IS2404* PCR with punch biopsy specimen and obtained a positivity ratio of 98% from 70 clinically diagnosed BUD patients [29]. Among 162 clinically diagnosed BUD patients with ulcerative lesions from Cameroon, 83% were confirmed by* IS2404* PCR [32]. In another study in Democratic Republic of Congo,* IS2404* PCR was used to diagnose 51 BUD patients with positivity ratio of 75% [6]. In a similar study in Ghana, DRB-PCR was used to clinically confirm 67% out of a cohort of 161 BUD patients. In this study, the positivity ratio for swab samples was 66%; analysis of tissue samples produced 57% positive results for ulcerative and 63% for nonulcerative lesions [24]. In another cohort study of 230 clinically diagnosed BUD patients from Ghana, DRB-PCR positivity ratios of 61% were determined for both swab and tissue samples [47].

In a related study in Togo, out of 202 suspected BUD cases, 109 BUD patients (54%) were PCR confirmed over a period of three years [28]. These findings indicate that PCR is considered the most sensitive method for the laboratory confirmation of BUD; however, protracted persistence of mycobacterial DNA in patients on antimycobacterial treatment makes PCR not applicable for monitoring of treatment success [17].

In an attempt to overcome the drawback of PCR, Beissner et al. developed a MU specific RNA-based viability assay combining a 16S rRNA reverse transcriptase real-time PCR (RT-qPCR) to determine bacterial viability with an* IS2404* quantitative real-time PCR (qPCR) for increased specificity and concurrent quantification of bacilli [36]. This technique has previously been applied for the detection of viable mycobacteria in patients with tuberculosis and leprosy [65, 66]. Conversely, the current test format requires well equipped laboratory with real-time PCR facilities and the costs per test limit its applicability. The reliance on PCR for diagnostic and research purposes in the field of BU requires the continued demonstration of its accuracy, reliability, and reproducibility. To this effect, Eddyani et al. established a multicenter external quality assessment program for PCR detection of BUD in clinical and environmental samples and reported an improved performance among participating laboratories [67].

### 3.6. Diagnostic Methods in Development

There is the need for simpler diagnostic that is both sensitive and specific and can be used at the point of care. The loop mediated isothermal amplification (LAMP) technique has previously been evaluated in many diseases, including malaria, and has been employed. The reported protocol employs four sets of primers, targeting sequences of the mycolactone encoding plasmid [27]. To overcome the requirement of cold-chains for transport and storage of reagents, Beissner et al. [68] recently establish a dry-reagent-based LAMP (DRB-LAMP) assay employing lyophilized reagents and clinically validated 140 clinical samples from 91 suspected BUD cases by routine assays, that is,* IS2404* dry-reagent-based (DRB) PCR, conventional* IS2404* PCR (cPCR), and* IS2404* qPCR, compared to cLAMP. Case confirmation and positivity rates of DRB-PCR or cPCR and cLAMP (62.64% and 52.86%) were comparable and there was no significant difference between the sensitivity of the assays (DRB-PCR and cPCR, 86.76%; cLAMP, 83.82%). Moreover, the sensitivity of cLAMP (95.83%) and the sensitivity of DRB-LAMP (91.67%) were comparable. However, all the reported studies used sophisticated equipment which cannot be employed in the field and there is the need for further work to use simpler equipment in low-resourced laboratory settings; moreover, obtaining purified DNA, as well as generating isothermal conditions, remains a major challenge for the use of the LAMP method under field conditions [33].

Another approach has been serological assays; however, currently available identified MU specific antigens such as the one detecting 85kda protein cannot differentiate between BU patients and exposed control individuals [69–71]. MUL-3720 protein has been identified as a promising target for antigen capture-based detection assays. It is highly expressed by MU and has no orthologs in other pathogenic mycobacteria. However, quest to use anti-MUL_3720 antibodies in a sandwich-ELISA format was found to be of insufficient sensitivity to make it suitable for the development of antigen capture-based diagnostic tests [72]. Thin layer chromatography for detecting mycolactone in clinical specimen has also been employed. TLC is comparatively simple but can be complicated by the presence of other lipids in the specimen. This step was informed by a study that demonstrated the presence of intact mycolactone in punch biopsies before and during antibiotic therapy using thin layer chromatography and mass spectrophotometry [73]. The group further provided proof of concept that indicated assays based on mycolactone detection in serum and ulcer exudates can form the basis of BU diagnostic tests. Fluorescent TLC had sensitivity of 73.2% and specificity of 85.7% when compared with PCR [68, 74]. A method using a boronate-assisted fluorogenic chemosensor in TLC was employed by Converse et al., to selectively detect mycolactone when visualized under UV light. They concluded that F-TLC may offer a new tool for confirmation of suspected clinical lesions and may be more specific than smear microscopy, faster than culture, and simpler than PCR [75]. Recently, Wadagni and colleagues evaluated fluorescent thin layer chromatography (fTLC) for detection of mycolactone in skin samples from patients with Buruli ulcer and compared them with samples from non-Buruli ulcer lesions that gave a negative result in the standard PCR test for MU [76]. However, further studies are needed to determine the feasibility of detecting mycolactone from samples obtained routinely.  Table 2 summarizes the various diagnostic techniques and their positivity ratios.

## 4. Conclusion and Future Perspective

Molecular techniques for the diagnosis of BUD have proven to be effective. Notably, real-time PCR offers a consistent quantitative and rapid tool for diagnosis and can be used for monitoring of treatment response of BUD. The development and application of reverse transcriptase PCR assays for the detection of viable MU would provide a valuable alternative for conventional mycobacterial cultures and thus considerably improve the clinical management of BUD. Culture remains the only method that detects viable bacilli. However, low sensitivity, long generation time and failure to distinguish between MU and other mycobacterial infections without extra confirmatory diagnostic tools, makes cultures unsuitable to support clinical management decisions timely. Furthermore, the application of molecular species identification assays, such as internal transcribed spacer length polymorphism or PCR restriction analysis of partial* rpoB* or* hsp*-65 genes [45, 55, 56, 63], would allow the distinction of MU from other nontuberculous mycobacteria. Most of these DNA-based techniques are present only in referenced and specialized centers. Conscious efforts should be channeled towards the formation of multicenter collaborative research programs. This will ensure reliability and reproducibility of test results and further allow validation, refinement, and adjustment of the application of molecular tools to specific clinical and epidemiological questions. The nonimmunogenic nature of mycolactone and other MU proteins have thwarted effort for serological assays. A general statement with respect to the performance of the various tests is not feasible since the positivity and sensitivity ratios are influenced by the quality of clinical diagnosis, duration of disease, pretreatment history of BUD patients, type and quality of diagnostic specimen and the duration of transport to the laboratory and transport conditions. It is evidenced that all currently available BU diagnostic techniques cannot be used as point of care and the need for a diagnostic test that can be used in the field cannot be overemphasized. Experimental studies on the use of aptamers against MU diagnostic target like mycolactone could be the key to the development of a point of care for BUD.

## Figures and Tables

**Table 1:** Summary of types of specimen and transport media for BU diagnosis.

Materials for diagnosis	Types	Country of origin	Reference
Specimen	Swabs	Ghana	Yeboah-Manu et al. [26]; de Souza et al. [27]
		Togo	Bretzel et al. [28]
	Punch biopsy	Ghana	de Souza et al. [27]; Phillips et al. [29]
		Australia	O'Brien et al. [30]
		Togo	Bretzel et al. [28]
		Benin	Ruf et al. [31]
	Biopsy	Ghana	Stienstra et al. [32]
	Fine needle aspirate	Ghana	Ablordey et al. [33]; Yeboah-Manu et al. [26]
		Togo	Bretzel et al. [28]
		Benin	Eddyani et al. [19]
	Whole bone or curetted bone samples	Ghana	Herbinger et al. [17]; Bretzel et al. [24]

Transport media	Modified Dubos medium (P5 medium)	Ghana	Stienstra et al. [32]; Yeboah-Manu et al., [34]
	Liquid Middlebrook 7H9 broth	Benin	Eddyani et al. [19]; Dobos et al. [35];
	10% OADC augmented with PANTA	Ghana	Wansbrough-Jones and Phillips [9]
	Solid transport media (STM)	Benin	Eddyani et al. [19]
	Liquid Nitrogen	Ghana	Rondini et al. [21]; Beissner et al. [36]

Decontamination methods	Oxalic acid	Ghana	Mensah-Quainoo et al. [37]; Yeboah-Manu et al. [34]
	N-Acetyl-cysteine-NaOH technique	Ghana	Schunk et al. [8]
	Reversed Petroff technique	Ghana	O'Brien et al. [30]
		Benin	Eddyani et al. [19]

DNA extraction method	Commercial	Ghana	de Souza et al. [27]
	In-house	Ghana	Ablordey et al. [33]
	Modified Boom DNA extraction procedure	Ghana	Durnez et al. [38]; Affolabi et al. [39]
	Commercial Maxwell 16 DNA extraction	Ghana	Affolabi et al. [39]
	One tube cell lysis (OT)	Ghana	Durnez et al. [38]
	FastPrep procedure	Ghana	Durnez et al. [38]

**Table 2:** Summary of various diagnostic techniques for BU.

Techniques	Number	+ve	−ve	Positivity ratio (%)	Geographic origin	Reference
Microscopy	39	23	16	58.9%	Australia	O'Brien et al. [30]
	31	7	24	22.5%	Benin	
	202	43	159	21.3%	Togo	Bretzel et al. [28]
	24	11	13	45.8%	Ghana	Beissner et al. [36]
	99	78	21	78.8%	Ghana	Mensah-Quainoo et al. [37]
	41	32	9	78.0%	Ghana	Yeboah-Manu et al. [34]
	44	15	29	34.1%	Ghana	Rondini et al. [21]
	65	19	46	29.2%	Benin/Ghana	Guimaraes-Peres et al. [57]
	164	38	126	23.2%	Cameroon	Noeske et al. [60]
	36	22	14	61.1%	DRC	Phanzu et al. [6]
	94	28	66	29.8	Ghana	Bretzel et al. [28]

Culture	33	—	33	—	Australia	O'Brien et al. [30]
	143	56	87	39.2%	Ghana	Stienstra et al. [32]
	41	32	9	78.0%	Ghana	Yeboah-Manu et al. [34]
	97	77	20	79.4%	Ghana	Mensah-Quainoo et al. [37]
	65	22	43	33.8%	Benin/Ghana	Guimaraes-Peres et al. [57]

Histopathology	12	12	—	100.0%	Benin	Ruf et al. [31]
	143	78	65	54.5%	Ghana	Stienstra et al. [32]
	36	27	9	75.0%	DRC	Phanzu et al. [6]

*IS2404 *PCR	30	21	9	70.0%	Ghana	Ablordey et al. [33]
	26	23	3	88.5%	Australia	O'Brien et al. [30]
	143	107	36	74.8%	Ghana	Stienstra et al. [32]
	202	109	93	54.0%	Togo	Bretzel et al. [28]
	24	18	6	75.0%	Ghana	Beissner et al. [36]
	65	55	10	84.6%	Benin/Ghana	Guimaraes-Peres et al. [57]
	162	135	27	83.3%	Cameroon	Noeske et al. [60]
	36	27	9	75.0%	DRC	Phanzu et al. [6]
	94	62	32	66.0%	Ghana	Bretzel et al. [28]

DRB-PCR	230	139	91	60.6%	Ghana	Siegmund et al. [47]

Real-time qPCR	18	15	3	83.3%	Ghana	Beissner et al. [36]
	44	29	15	65.9%	Ghana	Rondini et al. [21]
	74	44	30	59.5%	Benin	Affolabi et al. [39]

Nested PCR	21	21	0	100.0%	Ghana	Stienstra et al. [32]
	65	52	13	80.0%	Benin/Ghana	Guimaraes-Peres et al. [57]
	74	33	41	44.6%	Benin	Affolabi et al. [39]

*Others*						

LAMP assay	20	6	14	30.0%	Ghana	de Souza et al. [27]
	30	9	21	30.0%	Ghana	Ablordey et al. [33]
	20	13	7	65%	Ghana	de Souza et al. [27]

TLC	10	5	5	50.0%	Ghana	Sarfo et al. [73]

Serology	61	43	18	70.5%	Ghana	Dobos et al. [35]

Faecal	67	0	67	0.0%	Ghana	Sarfo et al. [74]
